# Streptavidin Modified ZnO Film Bulk Acoustic Resonator for Detection of Tumor Marker Mucin 1

**DOI:** 10.1186/s11671-016-1612-5

**Published:** 2016-09-13

**Authors:** Dan Zheng, Peng Guo, Juan Xiong, Shengfu Wang

**Affiliations:** 1 Hubei Collaborative Innovation Center for Advanced Organic Chemical Materials, Faculty of Physics and Electronic Science, Hubei University, Wuhan, 430062 China; 2 Faculty of Electronic and Engineering, Vocational College of WuHan Software Engineering, WuHan, 430205 China; 3 Hubei Collaborative Innovation Center for Advanced Organic Chemical Materials, Faculty of Chemistry and Chemical Engineering, Hubei University, Wuhan, 430062 China

**Keywords:** Thin-film bulk acoustic resonator, ZnO film, Resonant frequency, Biosensor, Mucin 1

## Abstract

A ZnO-based film bulk acoustic resonator has been fabricated using a magnetron sputtering technology, which was employed as a biosensor for detection of mucin 1. The resonant frequency of the thin-film bulk acoustic resonator was located near at 1503.3 MHz. The average electromechanical coupling factor ${K}_{\mathrm{eff}}^2$ and quality factor *Q* were 2.39 % and 224, respectively. Using the specific binding system of avidin-biotin, the streptavidin was self-assembled on the top gold electrode as the sensitive layer to indirectly test the MUC1 molecules. The resonant frequency of the biosensor decreases in response to the mass loading in range of 20–500 nM. The sensor modified with the streptavidin exhibits a high sensitivity of 4642.6 Hz/nM and a good selectivity.

**Electronic supplementary material:**

The online version of this article (doi:10.1186/s11671-016-1612-5) contains supplementary material, which is available to authorized users.

## Background

The piezoelectric biosensor has developed to a new type of biosensors, via the main electro-acoustic conversion devices, which can monitor the acoustic output signal of the transducer to reflect the physical and chemical changes of the measured substance [1–3]. At present, three main types of electroacoustic transducer are quartz crystal microbalance (QCM), surface acoustic wave sensor (SAW), and thin-film bulk acoustic resonator (FBAR). Among which, FBAR has the obvious merits such as high resonant frequency, small in size, high sensitivity, and compatibility with IC technology [4–8]. Nowadays, many work based on FBAR biosensor have been carried out and become the research hotspots in the fields of medical, food security, environment monitoring, etc. [9–11]. Gabl’s group detected label-free DNA and protein molecules based on integrated FBAR [12]. Mastromatteo et al. reported a highly sensitive mass sensor based on AlN/Si FBAR for artificial olfactory and bio-sensing application [13]. Chen et al. developed an acetylcholinesterase-coated shear mode FBAR for the detection of organophosphorus pesticides [14].

Mucin (MUC) is one kind of high molecular weight and glycosylated protein. The abnormal expression of MUC 1 is usually used as tumor markers in the clinical diagnosis of breast, prostate, pancreatic, colorectal, and lung carcinomas; therefore, monitoring the levels of MUC 1 is useful in clinical tumor diagnosis [15, 16]. But as far as we know, there are few reports about detection of MUC1 with streptavidin modified FBAR biosensor.

Biotin-Avidin system (BAS) is a new biological reacted amplified system. The non-covalent force in avidin-biotin system is known as the strongest non-covalent interaction at present, ten thousand times larger than the affinity between the antigen-antibody [17, 18]. BAS immunolabeling plays an important role in biosensor field due to their significant virtues including specificity, binding affinity, stability, and signal amplification [19].

In this work, FBAR is fabricated as a biosensors to detect MUC1 using the specific binding between biotin and avidin. The relationship between the resonant frequency shifts and MUC1 concentrations was investigated. The sensitivity of the biosensor using streptavidin active layer to detect MUC1 has been calculated.

## Methods

### Reagents and Materials

MUC1 (PDTRPAPGSTAPPAHGVTS APDTRPAPGSTAPPAHGVTSA) was purchased from GL Biochem corporation (Shanghai, China). MUC1 aptamers (5′-HS-(CH_2_)_6_-ACA CGG CAG TTG ATC CTT TGG ATA CCC TGG CGT GT-biotin-3′) were purchased from Sangon (Shanghai, China). Streptavidin was purchased from Promega (Sweden). HAuCl_4_ and tris (2-carboxyethy) phosphine hydrochloride (TCEP) were purchased from Sigma-Aldrich (USA). Deoxyadenosine triphosphate (dATP) was purchased from Aladdin (Shanghai, China). Mercaptopropionic acid, trisodium citrate, carbodiimide (EDC), *N*-hydroxysuccinimide (NHS) and other reagents were analytical grade. Phosphate buffer solution (PBS) and Tris-HCl buffer were pH 7.4 and prepared in laboratory.

### Fabrication of FBAR

**Fig. 1:**
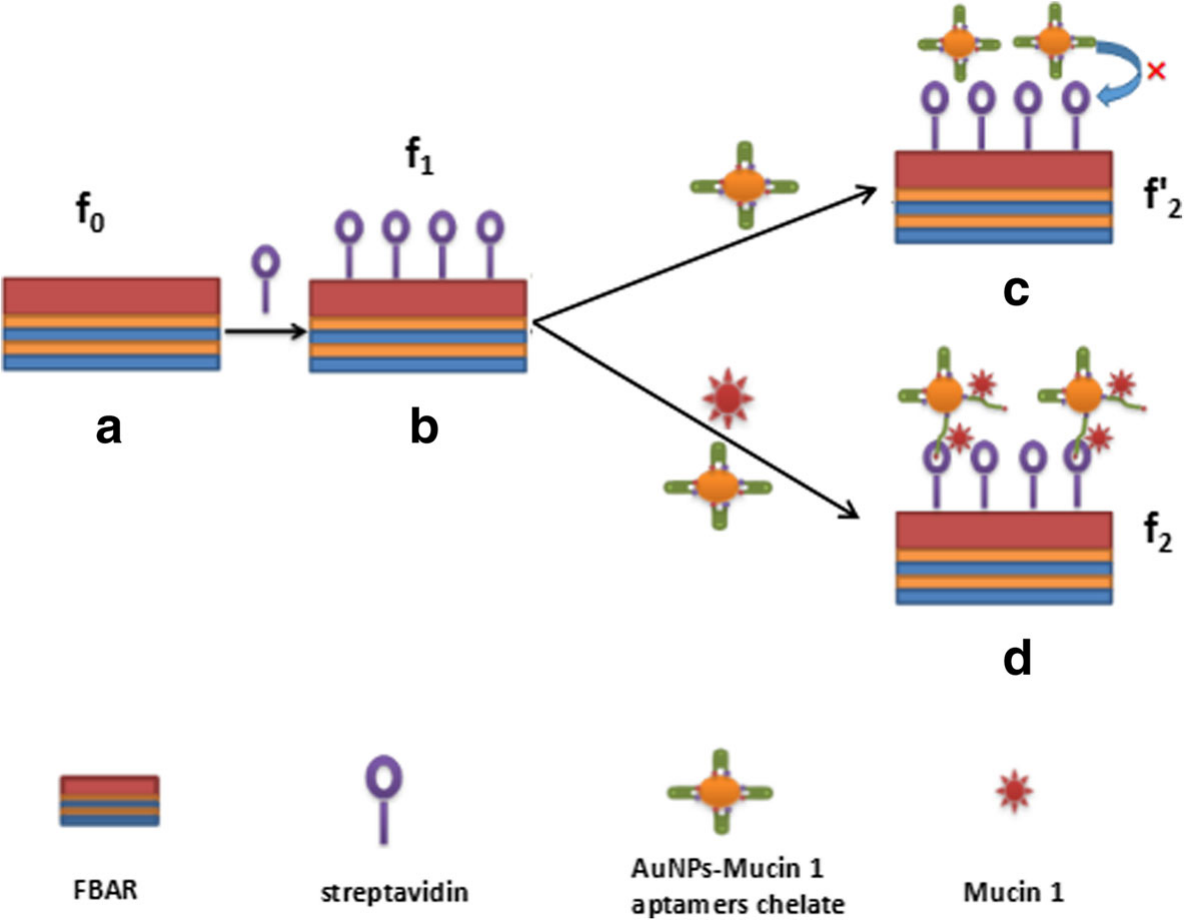
Schematic diagram of FBAR biosensor to detect MUC1 biomolecules. **a** The basic configuration of the ZnO-based FBAR. **b** Streptavidin was self-assembled on the active area of the FBAR as the sensitive layer. **c** In the absence of MUC1, the biotin is shielded in the AuNPs-MUC1 ampters chelates and cannot be captured by the streptavindin immobilized on FBAR. **d** When the target MUC1 is present, the immune binding between MUC1 and the thiol-biotin modified-aptamer reacts and makes the biotin exposed, as a result, the biotin can be captured by the streptavindin immobilized on FBAR

The basic configuration of the FBAR is shown in Fig. 1(a). There-period of Ti and Mo alternating layers was deposited on a p-type Si substrate using DC magnetron sputtering to create the Bragg acoustic reflector. The ZnO piezoelectric film was deposited onto the reflector using a RF reactive magnetron sputtering. The ZnO ceramic target was 99.99 % purity, 60 mm in diameter. The base vacuum of the cathode is no less than 2 × 10^−4^ Pa. The substrate temperature was kept at 300 °C. The argon/oxygen gas mixture was fixed at 6:1 and the working pressure was 0.5 Pa. A RF power of 150 W was deposited for 35 min. Au thin film of about 100 nm was served as the top electrode and patterned with a standard photolithography and lift-off process.

In order to precisely drop the analyte on sensor element, a polybenzoxazole (PBO) barriers with a height of several hundred micrometers were produced to separate the active area and the ground-single-ground (G-S-G) electrode using a second photolithographic technology.

### Self-Assembly Streptavidin as Sensitive Bio-Layer

As shown in Fig. 1(b), streptavidin were self-assembled on the active area of the FBAR for detection of MUC1 biomolecules. Firstly, the FBAR was successively sonicated with PBS solution, ethanol, and deionized water, followed by drying with nitrogen gas. Then, 2 mM mercaptopropionic acid was dropped in active zone and reacted for 1 h. Following, the resonator was washed with PBS solution and dried with nitrogen gas. And then, 3 μL of 400 mM EDC and 100 mM NHS mixed solution was added onto the active zone and kept for 1 h. After the resonator was thoroughly washed with PBS solution, 2 mg/mL streptavidin was manual dispensed onto the active zone and reacted for another 4 h. In the process, mercaptopropionic acid is an intermediate at one end which helps to anchor onto the gold electrode surface through Au-S bond, and the other end is bonded with streptavidin by condensation reaction. EDC/NHS is the catalyst in the carboxy or amino group condensation reaction. Finally, the resonator was washed with PBS solution and deionized water to remove the unbound streptavidin, and then dried with nitrogen gas.

### Indirect Method for Detection of MUC1

For amplifying the test signal, AuNPs-MUC1 ampters chelate was prepared. Heat 100 mL HAuCl_4_ solution (0.01 %) to boiling and add 2.7 mL trisodium citrate (1 %) for 10 min, stirring frequently, followed with washing, filtering, and drying, and after that we get the AuNPs. 1 mL AuNPs solution and 6 μL TCEP were added into 50 μL MUC1 ampters (10 μM), and then stirred for 2 h. The MUC1 aptamers were self-assembly on AuNPs via Au-S bonds and TCEP was the reducing agent in the reaction. Following, 100 μL of 14.1 μM dATP was added to the solution and stirred for 1 h. The dATP was served as a sealing agent to close the redundant sites on the AuNPs. The AuNPs-MUC1 ampters chelate was achieved after centrifugalizing. Lastly, the chelate was dissolved 1 mL of 0.01 M PBSB (PBS pH 7.4, 1 % BSA) solution, thus 500 nM AuNPs-MUC1 ampters solution has been prepared and stored at 4 °C for the following test. BSA can protect the activity of biological reagent even under ultra-low concentration.

For detection of MUC1 target molecules, MUC1 solution was mixed with the same volume of AuNPs-MUC1 ampters solution and incubated for 2 h at 37 °C. Then the mixture was introduced onto the FBAR’s active area and reacted with the self-assembly streptavidin for 30 min. Before measurement, the biosensor was washed with Tris-HCl buffer and dried with nitrogen gas. Various concentrations of MUC1 ranging from 25 to 500 nM were tested. To determine the selectivity of the modified biosensor, the frequency shifts of CA125, beta-Actin, CEA, and BSA were also monitored, and the concentration was the same value of 500 nM as MUC1 solution.

In this work, the special bonding of biotin and avidin was using for indirect detection of target MUC1. In the absence of MUC1, the biotin is shielded in the AuNPs-MUC1 ampters chelates and cannot be captured by the streptavindin immobilized on FBAR, as shown in Fig. 1(c). In Fig. 1(d), when the target MUC1 is present, the immune binding between MUC1 and the thiol-biotin modified-aptamer reacts and makes the biotin exposed. As a result, the biotin, along with the dually labeled chelate, is captured by the streptavindin sensitive layer. The frequency shifts of all-stage in the FBAR biosensor can be measured, and thus, the according biochemical reaction process can be judged.

### Testing Equipment

The morphologies of biomolecules and the cross section graph of the films were examined with field-emission scanning electron microscopy (SEM, JEOL 7100F). The crystal orientation of the FBAR was determined by X-ray diffraction (XRD, Bruker Advanced D8). The frequency characteristics of the biosensor were tested using the Agilent vector network analyzer (E5071C, USA) and RF probe station (PE-4, EverBeing, Taiwan)

## Results and Discussion

**Fig. 2:**
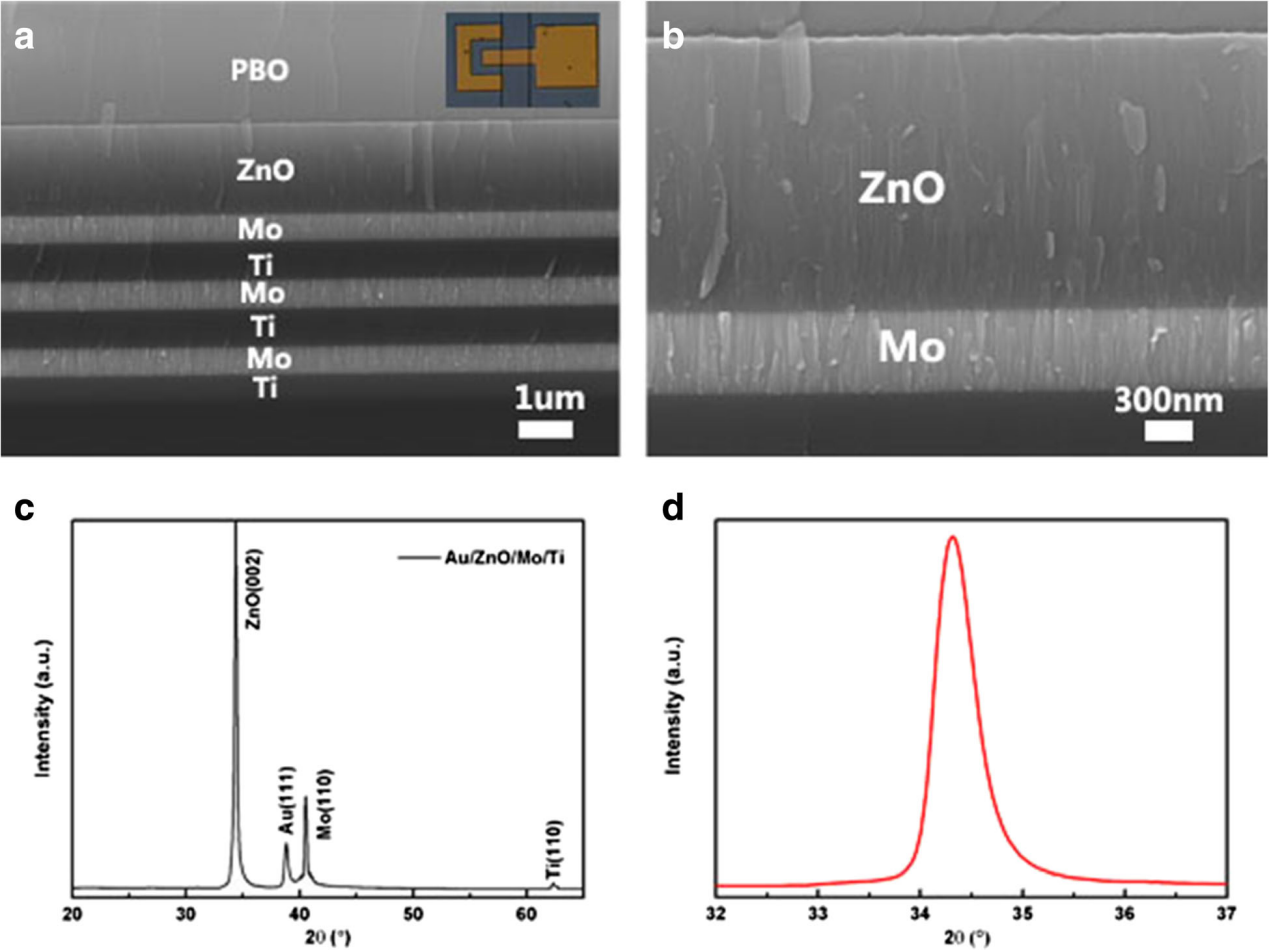
Characteristics of FBAR based on c-axis ZnO film: **a** the cross section scanning electron microscopy image, the insert is the G-S-G patterned Au electrode, **b** the amplification of ZnO piezoelectric film, **c** the XRD pattern of the device, and **d** the rocking curve of ZnO (002) peak

The basic composition of the device is depicted in the cross section SEM image as shown in Fig. 2a, which consists of a piezoelectric sandwich. The inset in Fig. 2a is the micrograph of one port ground-single-ground (G-S-G) patterned Au electrode with an active area of 200 × 200 μm^2^. PBO barrier layer has been prepared with a height of several hundred micrometers surrounding the active area for precisely manual dispensing. The magnified of the ZnO film is shown in Fig. 2b, in which highly c-axis oriented ZnO film with 1.5 μm thickness is clearly seen with a dense columnar structure. In Fig. 2c, (002) ZnO, (111) Au, (110) Mo, and (110) Ti are observed. The full-width half-maximum (FWHM) of the rocking curve of the ZnO (002) peak is very narrow, about 0.54° as shown in Fig. 2d, indicating preferred c-axis orientation corresponding with the wurtzite hexagonal structure.

**Fig. 3:**
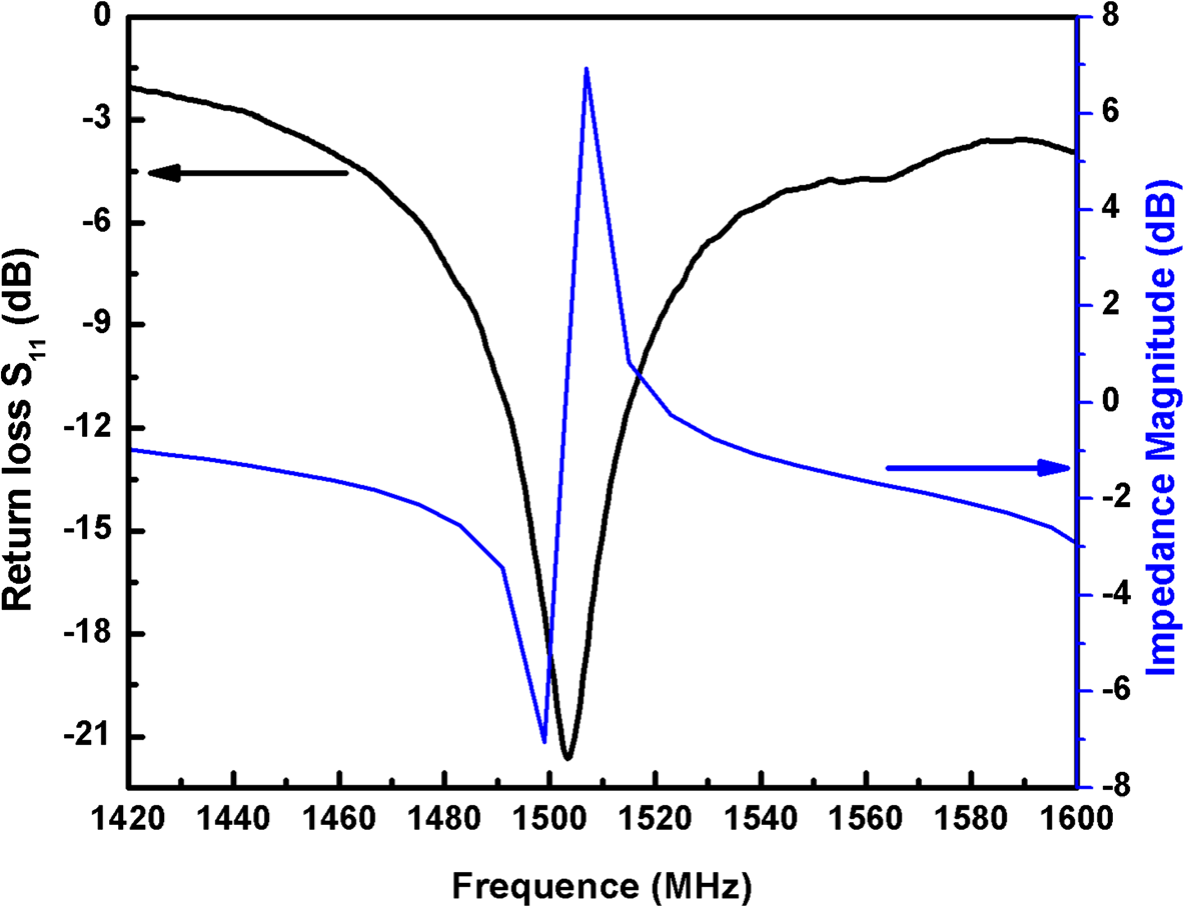
The amplitude of impedance and return loss of the bare FBAR device

Figure 3 shows the electrical response of the bare FBAR device. The resonant frequency (the minimum point of the return loss S_11_ curve) is recorded at 1503.3 MHz. The series and parallel resonant frequencies *f*_s_ and *f*_p_ were located at 1498.03 and 1512.67 MHz, respectively. The electromechanical coupling coefficient (${K}_{\mathrm{eff}}^2$) and the quality factor (*Q*) can be calculated using the following equations [20, 21]:

1 $$ {K}_{\mathrm{eff}}^2\left(\%\right)=\frac{\pi^2\left({f}_{\mathrm{p}}-{f}_{\mathrm{s}}\right)}{4{f}_{\mathrm{p}}}\times 100\ \%, $$

2 $$ Q=\frac{f_{\mathrm{m}}}{\delta {f}_{-3\  dB}}. $$

The measured average values of ${K}_{\mathrm{eff}}^2$ and *Q* were 2.39 % and 224, indicating a high performance of the prepared device.

**Fig. 4:**
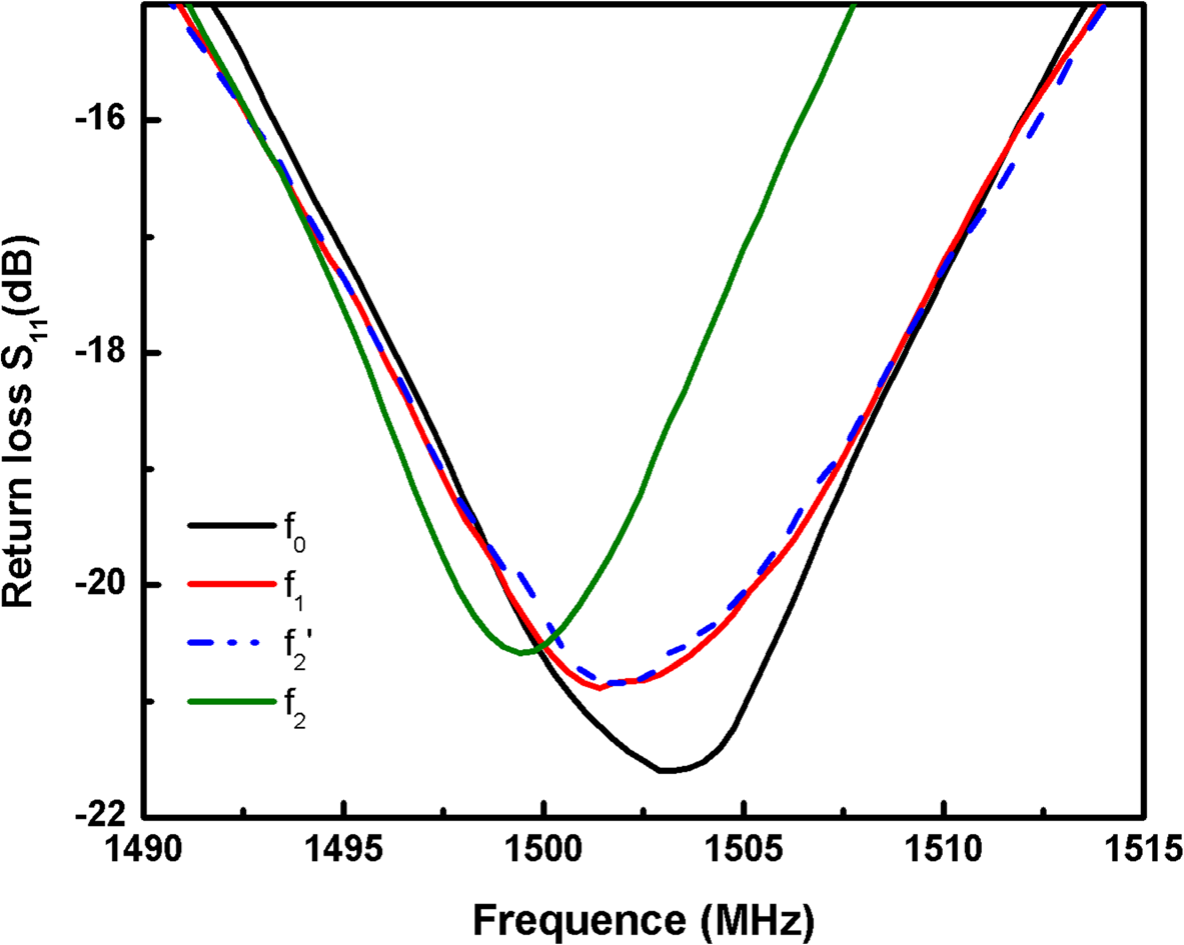
Comparison of the resonant frequency after every step of the reaction: the bare device *f*_0_, after streptavidin self-assembly on the FBAR *f*_1_, streptavidin-immobilized FBAR cannot bind with AuNPs-MUC1 ampters chelates without MUC1 target molecules *f*_2_′, and streptavidin-immobilized FBAR binding with AuNPs-MUC1 ampters chelates with MUC1 target molecules *f*_2_

As shown in Fig. 4, the resonant frequency *f*_0_ of the bare FBAR is 1503.3 MHz. After streptavidin self-assembly on the FBAR, a frequency shift *Δf*_1_ is calculated at around 1870.31 KHz. The resonant frequency was drifted down according to the FBAR’s theory that a small additional mass loading can result in a resonance frequency shift. After 500 nM AuNP-MUC1 aptamers chelates loaded on FBAR without the target MUC1, the frequency shift *Δf*_2_′ is 75.57 KHz. While after the same concentration of AuNP-MUC1 aptamers chelates loaded on FBAR with the target MUC1, the frequency shift *Δf*_2_ is 2653.06 KHz. Compared with the latter, the frequency shift of the former is negligible. When MUC1 is absent, the biotin is shielded in the “closed” AuNP-MUC1 aptamer chelates and cannot be captured by the streptavidin modified FBAR, therefore the value of *Δf*_2_′ is negligible reflecting chelates. However, when MUC1 is present, immune reaction between MUC1 and the MUC1 aptamer breaks down the “closed” AuNP-MUC1 aptamer chelates and makes the biotin exposed, resulting in the subsequent reaction between the biotin and streptavindin [22]. As a result, the frequency shift *Δf*_2_ is obvious, indicating that the biotin, along with the dually labeled chelates, is captured by the streptavindin modified FBAR. The testing results are according with the SEM morphologies of various biochemical phases as shown in Additional file 1: Figure S1. In Additional file 1: Figure S1(c), only few AuNPs-MUC1 aptamer chelates are found on the active area of the streptavidin modified FBAR when the target MUC1 is absent. While in Additional file 1: Figure S1(d), a large amount of AuNPs-MUC1 aptamer chelates are observed on streptavidin modified FBAR when the MUC1 marker is present. This proves the indirect method of using FBAR based on streptavidin-biotin immunoreaction to detect MUC1 tumor marker is feasible.

**Fig. 5:**
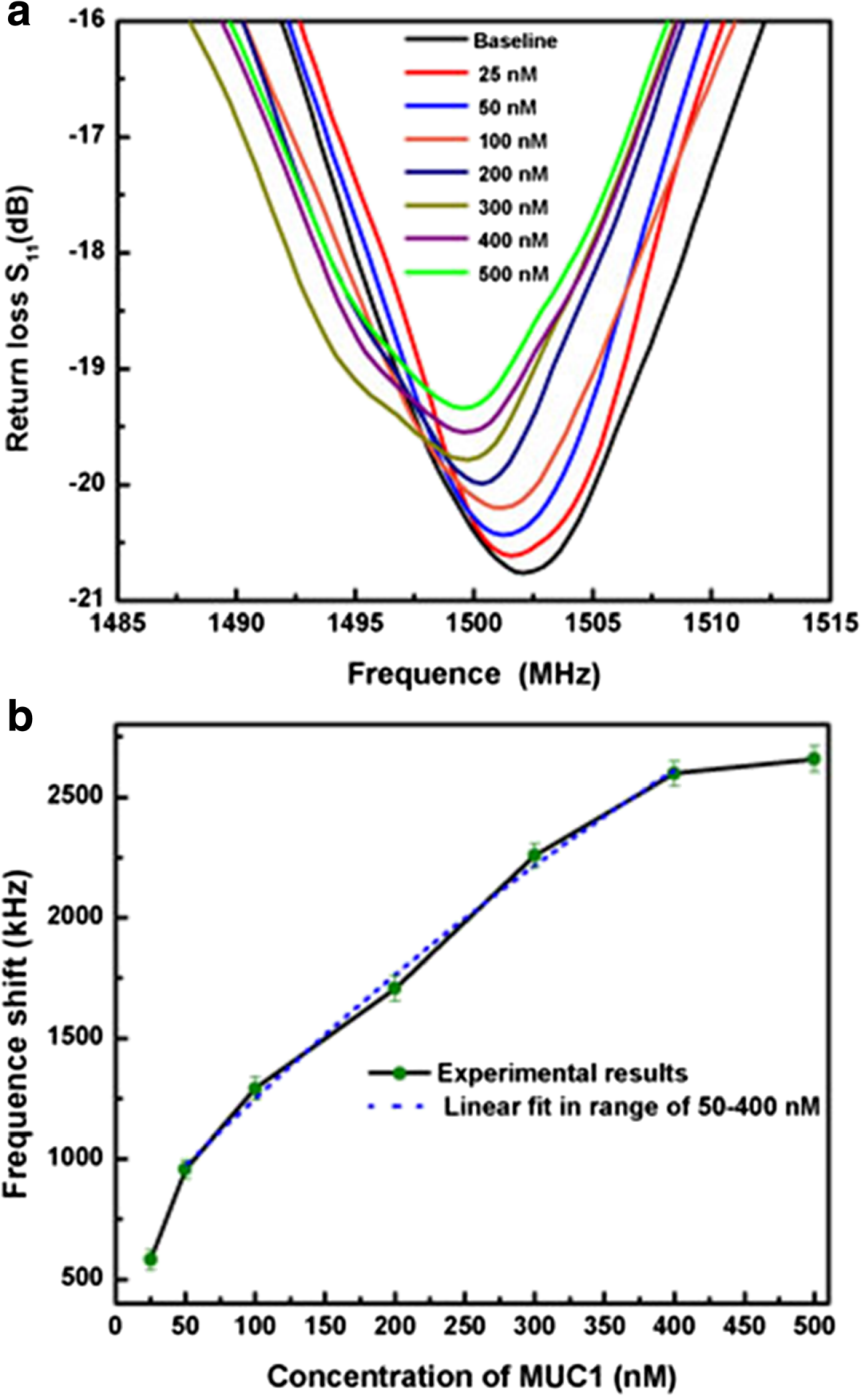
The frequency shifts of the biosensor to test various MUC1 concentrations. **a** The typical S_11_ responses of the streptavidin-immobilized FBAR to test various MUC1 concentrations from 20 to 500 nM, and **b** the relation curve between the resonant frequency shift and target MUC1 concentrations

Figure 5 shows the typical return loss S_11_ response of the streptavidin modified FBAR for detection of various MUC1 concentrations. In Fig. 5a, using streptavidin modified biosensor, the resonant frequency decreased with the mass load increase in the range of MUC1 concentrations from 20 to 500 nM. In Fig. 5b, the relationship between the resonant frequency and the increase of the MUC1 concentration in the range of 30–500 nM was nearly linear which complied with the Sauerbrey equation. The sensitivity of streptavidin modified FBAR is as high as 4642.6 Hz/nM which can be calculated from of the calibration curve. When the target MUC1 concentration was greater than 400 nM, the relationship between the resonant frequency and MUC1 concentration leveled off, indicating the binding between MUC1 and AuNPs-MUC1 aptamer chelates reached a maximum value.

In this work, the indirect method of using dually labeled thiol-biotin modified MUC1 aptamer to detect MUC1, the detection limit of MUC1 is as low as 20 nM and the sensitivity of the biosensor is as high as 4642.6 Hz/nM. As far as we know, the research of MUC1 based on FBAR is very few at present. Pang’s group reported an oxide-based fluorescent aptasensor for detection of MUC1 in a range of 0.04~10 mM [23]. Liu et al. presented MUC1 resonance energy transfer in a range of 64.9~1036.8 nM using electrochemiluminescence technology [24]. In this research, the good performance of the designed FBAR biosensor attribute to the strong bonding in the biotin-avidin system and the multiple magnifications. The non-covalent force in avidin-biotin system is known as the strongest non-covalent interaction at present, ten thousand times larger than the affinity between the antigen-antibody. On the other hand, every streptavidin can be bond with four biotins, which could realize the four times signal amplification [24]. In addition, the large molecular weight AuNPs can be captured by streptavidin self-assembly on the FBAR, together with the MUC1 and the aptamers, this will increase the frequency shift of device. As a result, the FBAR biosensor based on avidin-biotin system for detection of MUC1 has a good sensitivity.

**Fig. 6:**
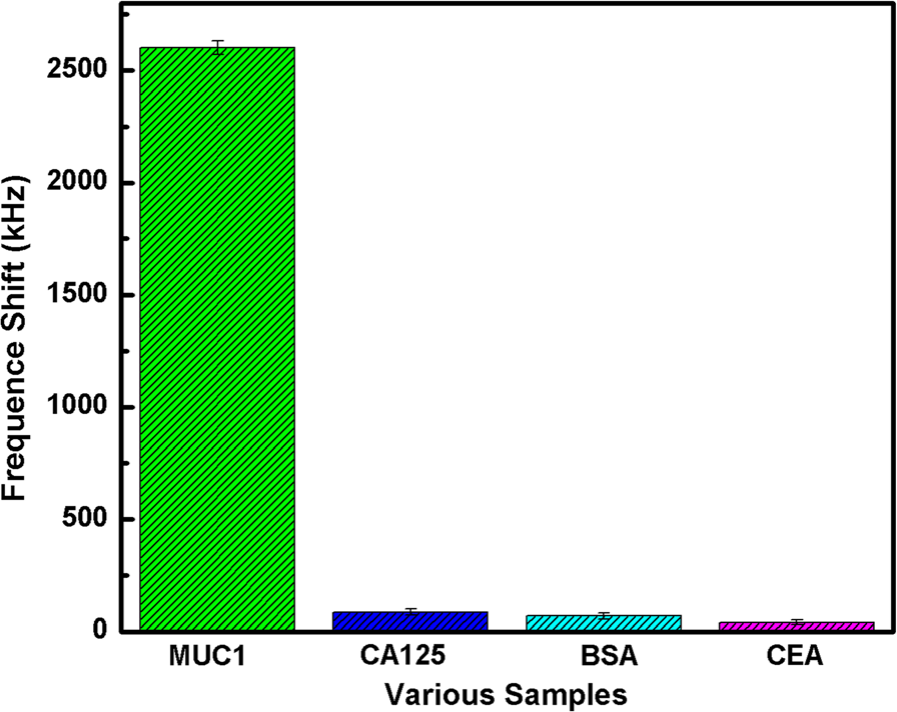
The frequency shift of streptavidin modified FBAR to detect MUC1, CEA, actin, BSA, and CA125. The testing molecules were fixed at the same concentration

For testing the selectivity of biosensor, a set of control experiments was carried out. As shown in Fig. 6, with streptavidin modified Au electrode, the fabricated FBAR was used to detect 500 nM carcinoembryonic antigen (CEA), Carbohydrate Antigen (CA125), Beta-Actin, Bovine Serum Albumin (BSA) and MUC1, respectively, some of which belong to the tumor mark family. Figure 6 showed that, MUC1 resulted in stronger frequency shift, while the other non-specific binding agents caused almost negligible frequency. The results indicate FBAR using streptavidin as sensitive lays has good selectivity to detect MUC1, shown the strong specific binding in biotin-avidin system and the application prospect.

## Conclusions

A ZnO-based film bulk acoustic resonator has been fabricated using a magnetron sputtering technology, which was employed as a biosensor for detection of MUC1. The resonant frequency of the FBAR was located near at 1503.3 MHz. The average electromechanical coupling factor ${K}_{\mathrm{eff}}^2$ and quality factor *Q* were 2.39 % and 224, respectively. Using the specific binding system of biotin-avidin, the streptavidin was self-assembled on the top gold electrode as the sensitive layer to capture the MUC1 molecules. The resonant frequency of the FBAR decreases in response to the mass loading in range of 20–500 nM. The FBAR modified with the streptavidin exhibits a high sensitivity of 4642.6 Hz/nM. The FBAR with streptavidin sensitive layer has high selectivity, sensitivity, and low detection limit, shown the promising prospect in biosensor field.

## Additional file

Additional file 1: Figure S1. SEM images after every step of the reaction: (a) streptavidin self-assembly on the FBAR on FBAR, (b) the prepared AuNPs-MUC1 ampters chelates, (c) few AuNPs-MUC1 ampters chelates captured by streptavidin without target MUC1, and (d) a large amount of AuNPs-MUC1 ampters chelates captured by streptavidin with target MUC1. (JPG 60 kb)
