# Supplementary figures and images for: Streptavidin Modified ZnO Film Bulk Acoustic Resonator for Detection of Tumor Marker Mucin 1
Source: Nanoscale Res Lett. 2016 Sep 13;11(1):396. doi: 10.1186/s11671-016-1612-5 (PMC5021656; doi:10.1186/s11671-016-1612-5)

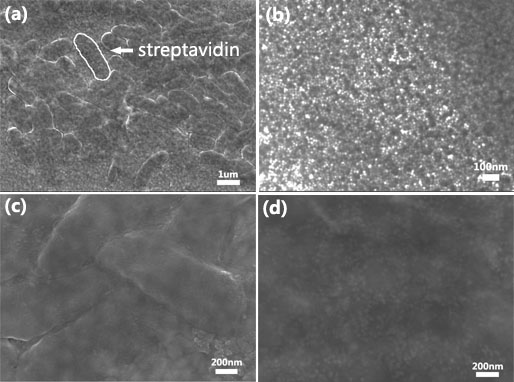

Supplement: Additional file 1: Figure S1. — SEM images after every step of the reaction: (a) streptavidin self-assembly on the FBAR on FBAR, (b) the prepared AuNPs-MUC1 ampters chelates, (c) few AuNPs-MUC1 ampters chelates captured by streptavidin without target MUC1, and (d) a large amount of AuNPs-MUC1 ampters chelates captured by streptavidin with target MUC1. (JPG 60 kb) [file 11671_2016_1612_MOESM1_ESM.jpg]
